# Plasma microRNA-320a as a Potential Biomarker of Physiological Changes during Training in Professional Volleyball Players

**DOI:** 10.3390/jcm11010263

**Published:** 2022-01-05

**Authors:** Rafał Podgórski, Marek Cieśla, Dominika Podgórska, Wojciech Bajorek, Artur Płonka, Wojciech Czarny, Robert Trybulski, Paweł Król

**Affiliations:** 1 Department of Biochemistry, Institute of Medical Sciences, Medical College of Rzeszow University, 35-959 Rzeszow, Poland; 2 Centre for Innovative Research in Medical and Natural Sciences, Medical College of Rzeszow University, 35-310 Rzeszow, Poland; 3 Department of Clinical Genetics, Institute of Medical Sciences, Medical College of Rzeszow University, 35-959 Rzeszow, Poland; ciesla_marek@wp.pl; 4 Department of Internal Diseases, Institute of Medical Sciences, Medical College of Rzeszow University, 35-959 Rzeszow, Poland; dpodgorska@ur.edu.pl; 5 Institute of Physical Culture Studies, Medical College of Rzeszow University, 35-959 Rzeszow, Poland; wbajorek@ur.edu.pl (W.B.); plonka77@op.pl (A.P.); wojciechczarny@wp.pl (W.C.); pkrol@ur.edu.pl (P.K.); 6 Department of Medical Sciences, The Wojciech Korfanty School of Economics, 40-659 Katowice, Poland; rtrybulski@o2.pl; 7 Provita Zory Medical Center, 44-240 Zory, Poland

**Keywords:** miRNA, athletes, training biomarkers, *IGF1R*

## Abstract

A deeper insight into the mechanisms responsible for athlete performance that may serve as specific and detailed training indicators is still desired, because conventionally used biomarkers provide limited information about the adaptive processes that occur during exercise. The objective of our study was to assess insulin-like growth factor 1 receptors (*IGF1R*) gene expression and evaluate plasma concentration of selected microRNAs (miRNAs) during a 10-week training period (sampling times: week 1, 4, 7, and 10) in a group of 12 professional female volleyball players. Circulating miRNAs (miR-223, miR-320a, and miR-486) with established concentration in plasma and documented association with the IGF1 signaling pathway, which is involved in muscle development and recovery, were tested. The levels of analyzed miRNAs, tested by one-way ANOVA, were significantly different between four training periods during a 10-week training cycle (miR-223 *p* < 0.0001, miR-320a *p* = 0.00021, miR-486 *p* = 0.0037, respectively). The levels of *IGF1R* also appeared to be different (*p* = 0.00092), and their expression showed a trend to increase between the first and third periods. In the fourth period, the expression decreased, although it was higher compared with the baseline. Correlations between concentration levels of miR-223 and miR-320a (rs = 0.54, *p* < 0.001), as well as between miR-320a and miR-486 (rs = 0.73, *p* < 0.001) were also found. In the fourth period, a negative correlation between miR-223 plasma level and leucocyte *IGF1R* expression was found (rs = −0.63, *p* = 0.028). Multiple linear regression analysis showed that miR-320a (*p* = 0.024) and creatine kinase (*p* = 0.028) had the greatest impact on the expression levels of the *IGF1R* gene. Future studies are required to define whether these miRNAs, especially miR-320a, as well as *IGF1R* expression could be useful biomarkers of physiological changes during exercise and to discover their detailed biological roles in mode-specific exercise training adaptations of professional athletes.

## 1. Introduction

The recognition of epigenetic mechanisms may serve as a new factor for better understanding, monitoring, and optimizing athlete performance during training and season periods. Optimizing the training process is related to the appropriate monitoring of anabolic and catabolic responses following different types of physical exercise. Many biomarkers of physical condition have been identified, such as creatine kinase (CK), lactic dehydrogenase, creatine kinase-MB isoenzyme, troponin T and NH_2_-terminal prohormone of brain natriuretic peptide, cortisol, and inflammatory marker—high-sensitivity C-reactive protein; however, all of them give only limited insight into exercise-induced adaptive processes and they do not render the full molecular changes arising during and after physical exercise [1,2,3]. Hence, new biomarkers capable of providing detailed knowledge about the changes in physical performance and evaluating exercise physiology are critically needed.

Insulin-like growth factor 1 (IGF-1) pathway is an evolutionary conserved regulatory module that is implicated in the metabolism of glucose, lipid, and protein [4]. IGF-1 affects almost every tissue in the human body by promoting cell proliferation, growth, and maturation through upregulation of anabolic processes [5,6]. Disruption of the IGF-1 signaling pathway has been associated with the onset of a variety of age-related diseases, including muscle disease, metabolic, cardiovascular, and neurodegenerative diseases, and cancer [7,8]. IGF-1 is also involved in muscle development, differentiation, regeneration and muscle mass increase, as well as inhibition of protein degradation [9,10]. IGF-1 acts by IGF-1 receptors that have a highly similar structure to that of insulin receptor [11]. The *IGF1R* gene occurs in large concentration in the skeletal muscle, liver, brain, and adipose tissue and plays a crucial role in IGF-1 pathway regulation [12,13]. Insufficient stimulation of IGF-1R due to low availability of its ligands is detrimental for the development of skeletal muscles. *IGF1R* is activated by ligand binding and initiation of receptor tyrosine kinase that leads to conformational changes of the receptor. This results in the activation of downstream signaling pathways of IGF-1, including Ras-mitogen-activated protein kinase pathway and phosphatidylinositol 3-kinase pathway, and finally, cell proliferation, differentiation, and survival [14]. *IGF1R* molecules are present on the surface of leucocytes, also known as cell differentiation marker CD221. They mediate communication within the immune system in the muscle regeneration process. Secretion of the IGF-1 and expression of its receptor in the blood by monocytes and granulocytes were identified as an important process related to muscle repair [15]. A role in *IGF1R*-related pathways in blood lymphocytes has also been confirmed. The combination of dihydrotestosterone and stimulation of IGF-1 affects cells adhesion, migration, and cytokines production, as well as modification expression of focal adhesion kinases [16]. Meta-analysis of the whole blood genes expression has shown that *IGF1R* gene is related to muscle strength [17]. Another study reported that *IGF1R* was overexpressed in the whole blood after high-intensity exercise in old champion athletes [18]. The study conducted by Abbasi et al. also confirmed the altered expression of *IGF1R* in the whole blood after intensive exercise [19]. MiRNAs inhibit *IGF1R* expression by directly targeting the 3′ untranslated region [6,12,20,21]. Due to its proliferation-promoting action, *IGF1R* has been investigated in many studies as a target in anticancer therapy [22,23].

Epigenetic mechanisms concern DNA methylation, histone modifications such as methylation, ubiquitination, phosphorylation, and acetylation, changes in higher-order chromatin structure, and a large variety of noncoding RNAs (ncRNAs). In the group of ncRNAs, long noncoding RNAs and small noncoding RNAs are distinguished [24,25]. The most important representatives of the second group are microRNAs (miRNAs) that range from 18 to 22 nucleotides in length mainly targeted to 3’-untranslated region of the gene [26]. In humans, more than 2500 miRNAs have been discovered that have been shown to play a fundamental role in regulation of gene expression at the post-transcriptional level by promoting mRNA degradation and inhibiting mRNA translation, which results in decreased expression levels of target proteins [27]. MiRNAs have been found in various human tissues and body fluids such as serum, plasma, cerebrospinal fluid, and urine, and they play crucial roles in a wide range of physiological and pathological processes [28,29]. MiRNAs that occur in biofluids, called circulating miRNAs, are transported to target cells within exosomes, microvesicles, and protein complexes and may derive from dead cells or be the products of cell secretion [30,31]. Circulating miRNAs are easily detectable, stable, and sensitive biomarkers that response superbly to metabolomic changes [32]. MiRNAs are stably secreted at rest in response to tissue damage and other pathological conditions [33,34]. Furthermore, miRNAs may also regulate muscle hypertrophy, as well as postexercise regeneration, and might be potentially a biomarker of overtraining and muscle fatigue, or an injury predictor [35,36,37].

The aim of the present study was to determine the expression of the *IGF1R* gene in the whole blood, as well as plasma concentration changes of selected miRNAs (miR-223, miR-320a, and miR-486) during a 10-week training period in a group of professional female volleyball players. All of the above-mentioned miRNAs are involved in regulation of IGF-1 signaling pathway [8,38,39,40]. We tried to find an easily accessible relatively noninvasive biomarker capable of providing a deeper insight into the physiological changes during training and that could be useful in determination of optimal training loads, as well as better recognition of the role of *IGF1R* in training adaptation, understood as body response related to the volume of exercise undertaken [41]. We assumed that *IGF1R* gene expression in total leucocytes and concentration of analyzed miRNAs in plasma would change within the study and show a constant trend as a response to the training program. To best of our knowledge, there is no study describing the influence of long-term training on miRNAs plasma quantity associated with *IGF1R* expression levels in professional athletes [42].

## 2. Materials and Methods

### 2.1. Subjects and Study Design

Blood samples were taken in the morning, on the same day of the week, after fasting, from the median cubital vein of 12 professional female volleyball players, who are multiple medalists of national and international competitions, at four time-points during a 10-week training cycle. Intervals between the sample collections were 3 weeks and coincided with the routine laboratory blood test schedule. The measurement at week 1 reflects period I, week 4 period II, week 7 period III, and week 10 period IV.

The participants had trained regularly for many years (13.8 years ± 6 months) before enrolling in our study. The average training experience of the study participants was 13 years and 8 months ± 6 years 5 months. The experiment was designed in such a way as to monitor the changes in sought biomarkers during the intensive preparation for a new season after a few weeks of nontraining during the summer break. The participants were not taking any medicines. The average age of the female players was 27 ± 5 years and 4 months (mean ± SD), the average height was 184.61 ± 9.37 cm, and the average body weight was 76.27 ± 12.76 kg. The Medical Ethics Committee the University of Rzeszow approved the study (protocol number 3/11/2017), and individuals provided written informed consents.

### 2.2. Training Cycle Characterization

The 10-week preparation period caused training effects assumed in the plan. No injuries that would have interrupted the players’ training cycle occurred during the preparation time.

The recording of training loads in the preparatory period based on the notation used by club coaches as part of the set training program consisted of 10 microcycles (weekly), in which 131 training units were carried out. The first 2 weeks were introductory, followed by a 6-week preparation period and finally, 2 weeks of specialist training. In the following training weeks, the percentage proportions of the type of training loads were as follows: weeks 1–3—aerobic effort (80%) and strength-resistance effort (20%); weeks 4–6—aerobic effort (60%) and resistance (40%); weeks 7–10—power-oriented training (80%), and 20% was aerobic in nature. During these periods, a total of 1320 min within 11 time units were allotted for biological regeneration and physiotherapeutic procedures in the preparatory period, such as classes at the pool, sauna, massage, and recovery pump. The time devoted to the implementation of 131 training units was 13,100 min, which amounted to 218.3 h. The highest training load time was planned in the 4th microcycle, in which 1800 min were carried out in 12 training units, and the smallest in the 8th microcycle, 780 min in 12 training units. Information about training loads and construction is presented in the Supplementary Files (Tables S1–S3, Figures S1–S3).

The maximum rate of oxygen (VO_2_ max), and levels of creatine kinase and cortisol in the blood were also regularly tested, as well as body composition parameters. VO_2_ max was calculated from the results obtained with the Beep Test [43].

Body composition analysis was performed using a Tanita BC-418 MA analyzer (Tanita Corporation, Tokyo, Japan), and the following parameters were recorded: height, body mass, fat mass in kilograms, percentage of body fat, total body water (TBW), fat-free mass in kilograms (FFM), basic metabolic rate (BMR), and body mass index (BMI). Cortisol and creatine kinase were determined using Alinity analyzer (Abbot, Abbott Park, IL, USA) by chemiluminescent microparticle immunoassay method and Alinity c creatine kinase reagent kit (Abbot, Abbott Park, IL, USA), respectively.

### 2.3. MicroRNAs Concentration Analysis

MicroRNAs were selected after reviewing current literature. Three miRNAs (miR-223, miR-320a, and miR-486) with an established concentration in plasma and documented association with the IGF1 signaling pathway were tested [8,38,39,40]. Blood samples with EDTA were collected and centrifuged at 3000 rpm for 10 min at 4 °C to separate plasma from cellular blood components. Plasma samples were aliquoted and stored at −80 °C until further analysis. After thawing, the samples were centrifuged at 3000× *g* for 5 min to pellet the cellular debris. MiRNAs were extracted from 200 µL of plasma using miRNeasy Serum/Plasma Advanced Kit (Qiagen, Hilden, Germany) according to the manufacturer’s instructions. At the beginning, 5.6 × 10^8^ copies of mimic cel-miR-39 (miRNeasy Serum/Plasma Spike-In Control, Qiagen, Hilden, Germany) and 1 µg of carrier RNA (MS2 RNA, Roche, Mannheim, Germany) were added to each sample. After extraction, the miRNAs samples were stored at −80 °C until downstream analysis. To perform the reverse transcription of miRNAs to complementary DNA (cDNA), the miRCURY LNA RT Kit (Qiagen, Hilden, Germany) was used according to the manufacturer’s recommendation. After that, cDNA was stored at −20 °C until expression analysis. Before polymerase chain reaction (PCR), cDNA was 30-fold diluted, and the reaction was performed using miRCURY LNA SYBR Green PCR Kit (Qiagen, Hilden, Germany) in the COBAS z480 Real Time PCR System under the thermal cycling conditions described in the mix manual. PCR was followed by a melt curve analysis. MiRNAs quantities were assessed by the following sets of primers: hsa-miR-486-3p miRCURY LNA miRNA PCR Assay; hsa-miR-320a miRCURY LNA miRNA PCR Assay; cel-miR-39-3p miRCURY LNA miRNA PCR Assay; product ID—QG-339306, Qiagen, Hilden, Germany. The expression levels of hsa-miR-223-3p were assessed by the primers designed by miRprimer software [44]. Primers had the following sequence (5′→3′): forward: GCCGCAGTGTCAGTTTGTCA, reverse: ACAGTTTTTTTTTTTTTTTGGGGTA, and were used at 450 nM final concentration. All samples were evaluated in duplicate. Due to the lack of consensus regarding which micro-RNA has a stable expression in plasma, the mimic cel-miR-39-3p was used for expression normalization [42,45,46,47,48]. Mimic cel-miR-39-3p was used for expression normalization. MiRNAs concentration levels were evaluated using the advanced relative quantification method using the maximum second derivative as the calculation model. Amplification efficiency for all targets was evaluated as previously described [49,50]. The expression results are presented as a normalized ratio.

### 2.4. IGF1R Expression Analysis

One milliliter of whole blood was used for *IGF1R* expression analysis. Erythrocytes were lysed using Lyse RBC 1 × buffer (Eurx, Gdansk, Poland). Briefly, 1 mL of blood was mixed with 4 mL of lysis buffer and left on ice for 10 min. After that, samples were centrifuged at 400× *g* for 10 min to pellet the cells. Leucocytes were washed with 5 mL of 1 × phosphate-buffered saline (PBS, Eurx, Gdansk, Poland) and centrifuged. Cells were washed a second time with PBS and centrifuged as described above. After that, leucocytes were mixed with 1 mL of RNA Extracol (Eurx, Gdansk, Poland) and stored at −80 °C until analysis. Total RNA was extracted by chloroform—isopropanol (Chempur, Piekary Slaskie, Poland) by a method previously described [51]. RNA pellets were resuspended in 15 µL RNase-free water. Then, 200 ng of RNA was reverse-transcribed using smART First Strand cDNA Synthesis Kit (Eurx, Gdansk, Poland) according to the manufacturer’s instruction in a final volume of 15 µL, using random hexamer primers. CDNA was stored at −20 °C until downstream analysis. Before PCR, cDNA was 2-fold diluted, and the reaction was performed using SG qPCR Master Mix (Eurx, Gdansk, Poland). The reaction was performed in the conditions given in the mix manual with annealing at 60 °C/30 s and extension at 72 °C/30 s. Melt curve analysis was performed after each reaction. PCR reaction was performed in 40 cycles in 10 µL of total volume. Expression of *IGF1R* gene was assessed by the following set of primers: (5’→3’): forward GCCGACGAGTGGAGAAATCTG and reverse TGGAGGTAGCCCTCGATCAC. For expression normalization, *GAPDH* gene was used, with the following primers sequences (5’→3’): forward AGAAGGCTGGGGCTCATTTG and reverse TGATGGCATGGACTGTGGTCAT. Primers were designed by the Primer-BLAST online tool [52]. The final concentration of all primers was 450 nM. Samples were evaluated in duplicate. Data analysis was performed as described above. PCR product specificity was evaluated by 1.5% agarose gel electrophoresis.

### 2.5. Statistical Analysis

Depending on the distribution, which was assessed by Shapiro–Wilk W test, the quantitative values with a normal distribution are presented as mean ± SD, otherwise as median with (25th–75th percentile). The miRNAs plasma concentration, *IGFR1* expression, as well as creatine kinase level were transformed using the logarithm function to obtain a normal or near-normal distribution. The body composition parameters were also transformed using the logarithm function, but only fat mass showed a normal distribution. Consequently, and because of an equal number of measurements between the periods, repeated-measures ANOVA and HSD Tukey’s post hoc tests were used to evaluate the differences in level of expression of each gene in the studied terms (matched repeats), as well as body composition parameters. The relationships between continuous variables were analyzed by Spearman’s rank correlation. Multiple linear regression analysis was performed to estimate the impact of miRNAs quantity and creatine kinase and cortisol on the expression levels of *IGF1R*.

A *p*-value less than 0.05 was considered statistically significant. The analyses were performed with STATISTICA version 13 (Dell Inc. 2016, Tulsa, OK, USA).

## 3. Results

**Figure 1:**
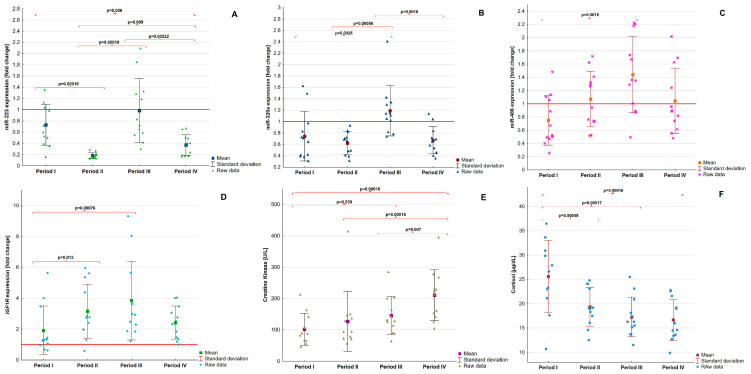
Differences between studied parameters during training periods. Abbreviations: *IGF1R*, insulin-like growth factor 1 receptor gene; miR, microRNA. A *p*-value was estimated by repeated-measures ANOVA. Statistically significant differences are in bold.

The levels of three miRNAs (miR-223, miR-320a, and miR-486) were significantly different between the training periods (*p* < 0.0001, *p* = 0.00021, and *p* = 0.0037, respectively). MiR-223 showed a variable quantity between the intervals. After the first period (baseline), the concentration was decreased; however, in the third period it increased and then in period IV, decreased again. MiR-320a showed only elevated concentration in the third period. MiR-486 level showed an increasing trend during the training, but only significant changes were observed between the first and third periods (*p* = 0.0018). The levels of *IGF1R* showed to be different between the trainings (*p* = 0.00092), and expression of the mentioned gene showed a trend to increase between the first and third periods. In the fourth period, its expression was decreased, although it was higher compared with the baseline. Creatine kinase levels increased during the training (*p* < 0.0001), opposite to cortisol levels, which decreased (*p* < 0.0001). The detailed results are presented in Table 1. The graphical representation of differences in mentioned variables between periods is shown in Figure 1.

The body composition parameters BMR, fat mass (%), FFM, and TBW changed during the training periods. Detailed results are presented in Table 2. The fat percentage and fat mass tended to decrease during the training, in opposition to FFM and TBW, which increased. Furthermore, BMR also increased during the study. A graphical representation of these data is shown in Supplementary Figures (Figures S4–S10).

Taking into consideration the whole training cycle (10-week period), the correlations between concentration levels of miR-223 and miR-320a (Spearman’s rank correlation coefficient (rs) rs = 0.54), as well as between miR-320a and miR-486 (rs = 0.73) were found. A negative correlation between miR-320a and cortisol (rs = −0.29), as well as between miR-486 and cortisol (rs = −0.49) was observed. No significant correlations between studied miRNAs and *IGF1R* expression were found. A negative correlation between *IGF1R* expression and BMI (rs = −0.37) was found. Detailed results are presented in Table 3.

In the first and second periods, no correlation between studied miRNAs and *IGF1R* and body composition parameters, as well as between CK and cortisol were found. In the third period, a strong negative correlation between *IGF1R* expression and BMI was observed (rs = −0.62). In the fourth period, a negative correlation between miR-223 plasma level and leucocyte *IGF1R* expression was found (rs = −0.63). Moreover, a positive correlation between miR-223 and CK (rs = 0.59) was found. Detailed results are presented in Supplementary Tables S4–S7.

Multiple linear regression analysis (R = 0.51; R2 = 0.26 and R2 (adjusted) = 0.17) showed that miR-320a (*p* = 0.024) and creatine kinase (*p* = 0.028) had the greatest impact on the expression levels of the *IGF1R* gene.

Detailed results of the analysis are presented in Supplementary Table S8.

After the preparatory period, all the participants improved their maximal oxygen uptake (VO_2_ max). The mean values of VO_2_ max rate in the group significantly increased from 42.22 mL/kg/min to 45.97 mL/kg/min (*p* < 0,00001) (Figure S11). Changes in the body composition parameters that were discovered by Tanita measurement after the training period include a decrease in fat mass and an increase in muscle mass with slight changes in body weight (Table 2).

## 4. Discussion

The objective of the present study was to determine the changes in levels of the circulating miR-223, miR-320a, and miR-486 and expression of *IGF1R* during a 10-week training period in a group of professional female volleyball players. The correlation analysis between circulating miRNAs changes with some biochemical as well as physical (CK, cortisol, BMI) parameters was also performed. It showed the potential of circulating miRNAs as possible biomarkers for assessing the exercise response.

The main finding of the study shows the alterations in the levels of all miRNAs and *IGF1R* expression within the training cycles, which reveals that miRNAs can be sensitive and reliable biomarkers of physical alteration. We have also shown that expression levels of miR-223 and miR-320a, as well as miR-320a and miR-486 were positively correlated during the whole training cycle. The miR-320 regulates glucose and lipid metabolism, as well as responses to oxidative stress-induced glycolysis [53,54]. Increased concentration of miR-320 in skeletal muscles reduces the lactate level, whereas its decreased concentration predisposes to the development of type II diabetes and may serve as a predictor biomarker of this condition because reduced circulating level of miR-320 precedes disease symptoms [55]. In a rat model of myocardial ischemia–reperfusion injury, the expression level of miR-320 is significantly upregulated and leads to mitochondrial apoptosis in cardiomyocytes [54,56]. MiR-320a regulates skeletal muscle mitochondrial metabolism and mitochondrial oxidative capacity [57]. MiR-486 is one of the muscle-specific miRNAs, called myomiRNAs, that positively regulates skeletal muscle growth, and its circulating level is altered during acute and chronic exercise. Decreased expression of miR-486 may be associated with metabolic changes during exercise and body adaptation induced by training [58,59]. This miRNA promotes myoblast differentiation and migration through the inhibition of Pax7 and Pax3 expression [60]. MiR-486 positively regulates the IGF-1/Akt pathway and myostatin signaling by targeting negative regulators of that process including phosphatase and tensin homolog, forkhead box O1, muscle RING-finger protein-1, and Atrogin1 [61,62]. Similar to miR-320a, it is involved in glucose metabolism, and both miRNAs were increased in insulin-resistant compared to insulin-sensitive individuals [38]. Involvement in similar biological processes may explain positive correlation between miR-320a and miR-486, as well as between miR-320a and miR-223. The last two miRNAs may precede thrombus formation in heart vessels resulting in acute myocardial infarction and may serve as useful tool to predict this condition [63]. A recent study has shown the level of miR-320a increased after 12 weeks exercise training in obese children and adolescents, which minimized risk of endothelial dysfunction and finally cardiovascular diseases. This correlation was not confirmed in our experiment, which might be explained by the difference between the compared groups [64].

We have also found relationships between the miR-320a level and creatine kinase and the expression levels of the *IGF1R* gene. Overexpression of miR-320a was reported to significantly downregulate the protein expression of *IGF1R* in cancer [39,65]. Studies on the direct influence of CK on *IGF1R* are scarce, but it seems likely that expression of *IGF1R* increases to counteract muscle damage, which is measured by higher serum CK concentration. It was indicated that only IGF1 and CK increased after a resistance exercise with different rest intervals [66,67]. Further examination of this issue is required, but the presented findings may suggest that miR-320a seems to be the most promising biomarker of training adaptation.

As we mentioned before, miR-223, miR-320a, and miR-486 are involved in the regulation of insulin-like growth factor 1 signaling pathway. Serum total IGF-1 levels increase with strength and endurance exercise [68,69], and the release of IGF-1 by macrophages plays an important role in recovery from muscle atrophy [70]. It has been also observed that VO_2_ max and physical activity correlate positively to resting plasma levels of IGF-1 [71,72]. Whereas, decreased IGF-1 levels were associated with various pathological conditions, including chronic diseases, such as muscle dystrophy, rheumatoid arthritis (RA), inflammation, and malnutrition [73,74,75]. Fragala and colleagues have reported that resistance exercise seems to increase *IGF1R* expression on monocytes and granulocytes, and suggested that IGF-1 may contribute to the communication with immune cells and muscle tissue regeneration [15]. It has been also shown that inflammation is directly associated with a high expression of *IGF1R* in leukocytes [76] and with systemic inflammation expansion of the inflamed synovia in RA patients [75]. Another study suggested a positive impact of progressive resistance training on enhancement of *IGF1R* density in elderly human skeletal muscle and markers of tissue regeneration [77]. This may explain the fact that the expression of leukocyte *IGF1R* remained at a more or less consistently elevated level compared to its initial value to counteract muscle tissue damage. *IGF1R* is also a factor in muscle hypertrophy induced by androgen receptor stimulation after resistance and endurance trainings [78]. Previous reports have shown *IGF1R* in the whole blood was differentially expressed after intensive exercise. In the presented study, we indicate changes in *IGF1R* gene in relation to long-term training. It is necessary to mention that we evaluated the expression in total blood leucocytes due to the fact that altered concentration of this molecule had been reported on monocytes, granulocytes, and lymphocytes and that blood is an easily accessible material to use in cyclical analyses [15,16]. The wide range of expression in major subpopulations of white blood cells allows to use this molecule as a peripheral marker of training. An additional advantage is that *IGF1R* expression in blood can be associated with intensive exercise [18,19] and muscle strength [17]. It is also proven that suppression of *IGF1R* by natural autoantibodies impairs physical strength [79].

MiRNAs have been reported as potential extracellular biomarkers of various conditions and may be easily evaluated in body fluids such as plasma, serum, urine, and saliva [29]. There is growing evidence showing that microRNAs significantly impact muscle growth, regeneration, and metabolism [80]. MicroRNAs have a great potential to become diagnostic and/or prognostic markers. Their separation from cellular ribonucleases protects them against rapid degradation and causes miRNAs to have stable concentration, making them suitable as biomarkers [48]. In addition, miRNAs in plasma are noninvasive and inexpensive to quantify. Furthermore, PCR is a highly specific and sensitive method, which means cycle amplification can be used as a powerful tool for evaluating new biomarkers [29]. We have found that quantification of circulating miRNAs in plasma may be a more desirable marker than the assessment of the level of transcripts (messenger RNA) due to the fact that it is a less complicated methodology and has a stable expression of miRNAs in biofluids. Evaluation of messenger RNA (mRNA) should be normalized (in the first step of normalization) on total blood count and proportion of white blood cell subpopulations, which causes some inconvenience. Circulating biomarkers can be simply normalized by sample volume. Thus, initial determination of the associations between these molecules and targeted transcripts is necessary, which is shown in our study. Moreover, quantitative changes in mRNA expression require the presence of a reference gene (comparative method). The quantity of miRNAs can be evaluated easily by standard curve method with the use of mimic miRNAs as a standard. This approach can increase the precision and comparability of the measurements, and may reduce the costs.

Moreover, we observed a negative correlation between cortisol and miR-320a, as well as miR-486. Resting cortisol level generally reflects a long-term training stress. Cortisol plays a crucial role in regulation of energy homeostasis and metabolism. It reveals catabolic functions and has great impact on type II muscle fibers. During and after acute exercise, cortisol concentration increases as a result of adaptation to the efforts that counteract the cytokine synthesis, muscle inflammation, and damage [81,82]. Long-term training does not appear to produce consistent patterns of cortisol secretion, but chronically elevated cortisol levels impair anabolic processes and decrease skeletal IGF-I synthesis by reducing IGF-I transcript levels [83,84,85,86].

We have also indicated that CK increased continuously during the training period and in the fourth period was positively correlated with miR-223. This miRNA is a well-known proinflammatory molecule that promotes skeletal muscle regeneration by regulating inflammation [87]. It is also involved, inter alia, in the regulation of hematopoiesis, glucose uptake by cardiomyocytes, and cholesterol uptake [88,89,90]. Recent studies reveal that miR-223 is significantly upregulated in the early stage of muscle regeneration after skeletal injury, which explains the positive correlation with the increased value of CK—the marker of muscle destruction [87]. This suggests that a high level of miR-223 in plasma may be associated with muscle destruction and potential injury. Recently, negative correlation between mir-223 and *IGF1R* expression has been observed, which may suggest that exercise-related inflammation is reduced due to lower resistance training load.

The participants’ exercise and fitness capacities were improved after the 10-week training program. The studies conducted so far indicate that the results achieved in Beep Test have a moderate-to-high accuracy in estimating VO_2_ max (rp = 0.66–0.84) and are higher when other variables are taken into consideration, such as gender, age, and body weight (rp = 0.78–0.95). Beep Test results (indirect and noninvasive test) are strong indicators of cardiorespiratory performance in adults [91]. Physical exercise affect many signaling pathways that influence energy metabolism, inflammation, regeneration, and remodeling of myocardial and skeletal muscles [92,93]. Performance volleyball is characterized by intermittent exercise with repeated and repetitive episodes of high-intensity and short-duration effort. The energy demand of volleyball comes from both aerobic and anaerobic energy systems. These features of volleyball make the signature of circulating miRNAs different from that of other aerobic and anaerobic exercise types.

Exercise adaptation is a very complex process, and it is not easy to find a distinct factor that could reflect accurately the physiological changes during training. There are numerous genes, more than 200, involved in muscle growth and training adaptation [94]. Alterations in gene expression occur in response to multiple stimuli associated with adaptation of the biological system to exercise training. Skeletal and cardiac muscles contraction results in releasing of various signaling kinases and the activation of many molecular processes mediating oxidative and nonoxidative metabolism and angiogenesis. In addition, gene expression can be altered via epigenetic mechanisms, such as DNA methylation, histone acetylation and phosphorylation, and micro-RNAs, which can alter the gene expression [95,96,97]. It has been also proven that repeated exercise stimuli cause longer-lasting effects on gene transcription and protein expression compared to an acute, single exercise bout [98,99].

We are aware that our study, like any other initial study, has several limitations. First, the number of tested athletes should be increased. Alterations caused by, for example, disease, inflammation, or menstruation in one of the participants may disrupt the proper interpretation of the results. Second, to observe the difference of exercise abilities between female and male, it would be interesting to investigate the miRNAs and *IGF1R* expression changes in response to long-term training by using also male athletes as subjects. Third, the data that could describe in detail the athletes’ physical performance, such as vertical jump ability, agility, sprint ability, and anaerobic capacity, at each training cycle were not acquired. Four, the presented study was restricted to volleyball players. Future studies are required to determine whether the changes of sought biomarkers are applicable to other similar sport disciplines. Finally, quantitative analysis was limited to a subset of the relevant miRNAs involved in the regulation of IGF1, and high-throughput screening is needed to acquire a more complete profile of circulating miRNAs within a long-term volleyball training.

## 5. Conclusions

Long-term exercise alters the plasma miRNAs levels and *IGF1R* expression in volleyball athletes, which may provide significant information about the physical and molecular condition of sportspersons. Our study describes an overall effect of exercise on circulating miRNAs profiles in plasma and *IGF1R* expression of highly trained women. MiR-320a seems to be the most valuable molecule for further investigation due to its association with *IGF1R* expression, positive correlation with other studied miRNAs, and relationship with creatine kinase and cortisol. Future studies are required to define whether these miRNAs, especially miR-320a, as well as *IGF1R* expression could be useful biomarkers of physiological changes during exercise and discover their detailed biological roles in mode-specific exercise training adaptations of professional athletes. 

## Figures and Tables

**Table 1:** Differences in targets expression levels and laboratory parameters between studied periods.

Parameter	Period I	Period II	Period III	Period IV	*p*-Value
miR-223	0.73 ± 0.37	0.18 ± 0.05	0.98 ± 0.57	0.37 ± 0.18	**<0.0001**
miR-320a	0.74 ± 0.43	0.62 ± 0.2	1.19 ± 0.44	0.67 ± 0.24	**0.00021**
miR-486	0.75 ± 0.38	1.07 ± 0.42	1.44 ± 0.58	1.04 ± 0.49	**0.0037**
*IGF1R*	1.91 ± 1.57	3.15 ± 1.76	3.84 ± 2.55	2.41 ± 1.08	**0.00092**
CK (U/L)	101.82 ± 51.12	126.92 ± 96.27	145.25 ± 61.12	210.58 ± 80.91	**<0.0001**
Cortisol (µg/dL)	25.57 ± 7.45	19.36 ± 3.97	17.27 ± 4.09	16.66 ± 4.26	**<0.0001**

Data are presented by mean ± SD. Abbreviations: CK, creatine kinase; *IGF1R*, insulin-like growth factor 1 receptor gene; miR, microRNA. A *p*-value was estimated by repeated-measures ANOVA. Statistically significant differences are in bold.

**Table 2:** The differences in body composition parameters between studied periods.

Body Composition Parameter	Period I	Period II	Period III	Period IV	*p*-Value
Weight, kg	77.3 ± 10.08	77.83 ± 10.12	77.98 ± 10.61	78.17 ± 10.49	0.064
BMI, kg/m^2^	22.68 ± 2.18	22.8 ± 2.21	22.83 ± 2.22	22.85 ± 2.18	0.33
Fat, %	20.83 ± 3.77	19.38 ± 3.08	19.14 ± 3.71	18.66 ± 3.58	**0.00001 ^A^**
Fat mass, kg	16.33 ± 4.76	15.28 ± 4.22	15.09 ± 4.73	14.89 ± 4.7	**0.00048 ^B^**
FFM, kg	60.98 ± 6.38	62.56 ± 6.57	62.96 ± 6.64	63.53 ± 6.85	**<0.00001 ^C^**
TBW, kg	44.63 ± 4.66	45.81 ± 4.81	46.16 ± 4.9	46.45 ± 5.05	**<0.00001 ^D^**
BMR, kJ	7568.75 ± 848.73	7740.33 ± 881.37	7835.58 ± 959.55	7828.5 ± 910.59	**0.00015 ^E^**

Body composition data are presented as mean ± SD. Abbreviations: BMI, body mass index; BMR, basic metabolic rate; FFM, fat-free mass; TBW, total body water. A *p*-value was estimated by repeated-measures ANOVA. Statistically significant differences are in bold. The time point differences were as follows: **^A^**: differences were found between period I and period II (*p* = 0.002), between period I and period III (*p* = 0.0004), and between period I and period IV (*p* = 0.0002). **^B^**: differences were found between period I and period II (*p* = 0.037), between period I and period III (*p* = 0.003), and between period I and period IV (*p* = 0.0006). **^C^**: differences were found between period I and period II (*p* = 0.0008), between period I and period III (*p* = 0.0002), and between period I and period IV (*p* = 0.0002). **^D^**: differences were found between period I and period II (*p* = 0.0014), between period I and period III (*p* = 0.0002), and between period I and period IV (*p* = 0.0002). **^E^:** differences were found between period I and period II (*p* = 0.028), between period I and period III (*p* = 0.0005), and between period I and period IV (*p* = 0.0006).

**Table 3:** Spearman’s rank correlation between studied markers in the whole training period.

	miR-223	miR-320a	miR-486	*IGF1R*	WEIGHT	BMI	BMR	FAT%	FAT MASS	FFM	TBW	CK	Cortisol
**miR-223**	1	**0.54** (*p* < 0.001)	0.06	−0.16	−0.19	−0.03	−0.16	−0.11	−0.18	−0.18	−0.18	0.004	0.02
**miR-320a**	**0.54** (*p* < 0.001)	1	**0.73** (*p* < 0.001)	0.2	0.03	−0.12	0.06	0.02	−0.01	0.06	0.05	0.09	**−0.29** (*p* = 0.048)
**miR-486**	0.06	**0.73** (*p* < 0.001)	1	0.23	0.17	−0.09	0.21	0.03	0.09	0.21	0.2	0.05	**−0.49** (*p* < 0.001)
***IGF1R***	−0.16	0.2	0.23	1	−0.13	**−0.37** *p* = 0.01	−0.09	−0.1	−0.08	−0.06	−0.06	−0.19	−0.23

Statistically significant values of Spearman’s rank correlation coefficients (r_s_) are bold, and the *p*-values are provided. In cases without the *p*-values, the r_s_ coefficient represents insignificant data. Abbreviations: BMI, body mass index; BMR, basic metabolic rate; CK, creatine kinase; FFM, fat-free mass; *IGF1R*, insulin-like growth factor 1 receptor gene; miR, micro-RNA; TBW, total body water.

## Data Availability

The datasets used and/or analyzed during the current study are available from the corresponding author on reasonable request.

## Supplementary Materials

The following are available online at https://www.mdpi.com/article/10.3390/jcm11010263/s1, Figure S1: Time volume (minutes) in respective microcycles; Figure S2: The volume of training load (kilograms) for the team in respective microcycles; Figure S3: Total distance (kilometers) covered by the team in respective microcycles; Figure S4: The changes in weight between training periods; Figure S5: The changes in body mass index between training periods; Figure S6: The changes in percentage of fat between training periods; Figure S7: The changes in fat mass between training periods; Figure S8: The changes in fat-free mass between training periods; Figure S9: The changes in total body water between training periods; Figure S10: The changes in basic metabolic rate between training periods; Figure S11: The changes in VO_2_ max rate between first and last training period. (Statistical differences were calculated by the t-test for dependent samples.); Table S1: Dates and number of training units carried out in the preparatory period; Table S2: The volume of training load (kilograms) for the team in respective microcycles; Table S3: Average distances covered by players during the preparation period (km); Table S4: The Spearman’s rank correlation between studied markers in the first training period (baseline); Table S5: The Spearman’s rank correlation between studied markers in the second training period; Table S6: The Spearman’s rank correlation between studied markers in the third training period; Table S7: The Spearman’s rank correlation between studied markers in the fourth training period; Table S8: The multiple linear regression analysis using insulin-like growth factor 1 receptor gene expression levels as outcome.
