# Supplementary material for: Plasma microRNA-320a as a Potential Biomarker of Physiological Changes during Training in Professional Volleyball Players
Source: J Clin Med. 2022 Jan 5;11(1):263. doi: 10.3390/jcm11010263 (PMC8746094; doi:10.3390/jcm11010263)
Supplement: Supplementary file 1 [file jcm-11-00263-s001.zip › jcm-1427184-supplementary.pdf]

Journal of *Clinical Medicine*

MDPI

Supplementary Material

Plasma microRNA-320a as a Potential Biomarker of Physiological Changes During Training in Professional Volleyball Players

Rafał Podgórski [1,2,*], Marek Cieśla [3], Dominika Podgórska [4], Wojciech Bajorek [5], Artur Płonka [5], Wojciech Czarny [5], Robert Trybulski [6,7] and Paweł Król [5]

1 Department of Biochemistry, Institute of Medical Sciences, Medical College of Rzeszow University, Rzeszów, Poland, rpodgorski@ur.edu.pl
2 Centre for Innovative Research in Medical and Natural Sciences, Medical College of Rzeszow University, Rzeszów, Poland,
3 Institute of Medical Sciences, Medical College of Rzeszow University, Rzeszów, Poland; ciesla_marek@wp.pl
4 Department of Internal Diseases, Institute of Medical Sciences, Medical College of Rzeszow University, Rzeszów, Poland; dpodgorska@ur.edu.pl
5 Institute of Physical Culture Studies, Medical College of Rzeszow University, Rzeszów, Poland; wbajorek@ur.edu.pl (W.B.); plonka77@op.pl (A.P.); wojciechczarny@wp.pl (W.C.); pkrol@ur.edu.pl (P.K.)
6 Department of Medical Sciences, The Wojciech Korfanty School of Economics, Katowice, Poland; rtrybulski@o2.pl
7 Provita Zory Medical Center, Zory, Poland
* Correspondence: rpodgorski@ur.edu.pl; Tel.: +48-17851-68-55

Detail training description

Table S1. Dates and number of training units carried out in the preparatory period.

Microcycle number	Dates	Training units
**Microcycle 1**	**15-19.07.2020**	**11**
**Microcycle 2**	**20-26.07.2020**	**11**
**Microcycle 3**	**27.07-2.08.2020**	**14**
**Microcycle 4**	**3.08-9.08.2020**	**12**
**Microcycle 5**	**10-16.08.2020**	**11**
**Microcycle 6**	**17-23.08.2020**	**16**
**Microcycle 7**	**24-30.08.2020**	**16**
**Microcycle 8**	**31.08-6.09.2020**	**12**
**Microcycle 9**	**7-13.09.2020**	**14**
**Microcycle 10**	**14-19.09.2020**	**14**

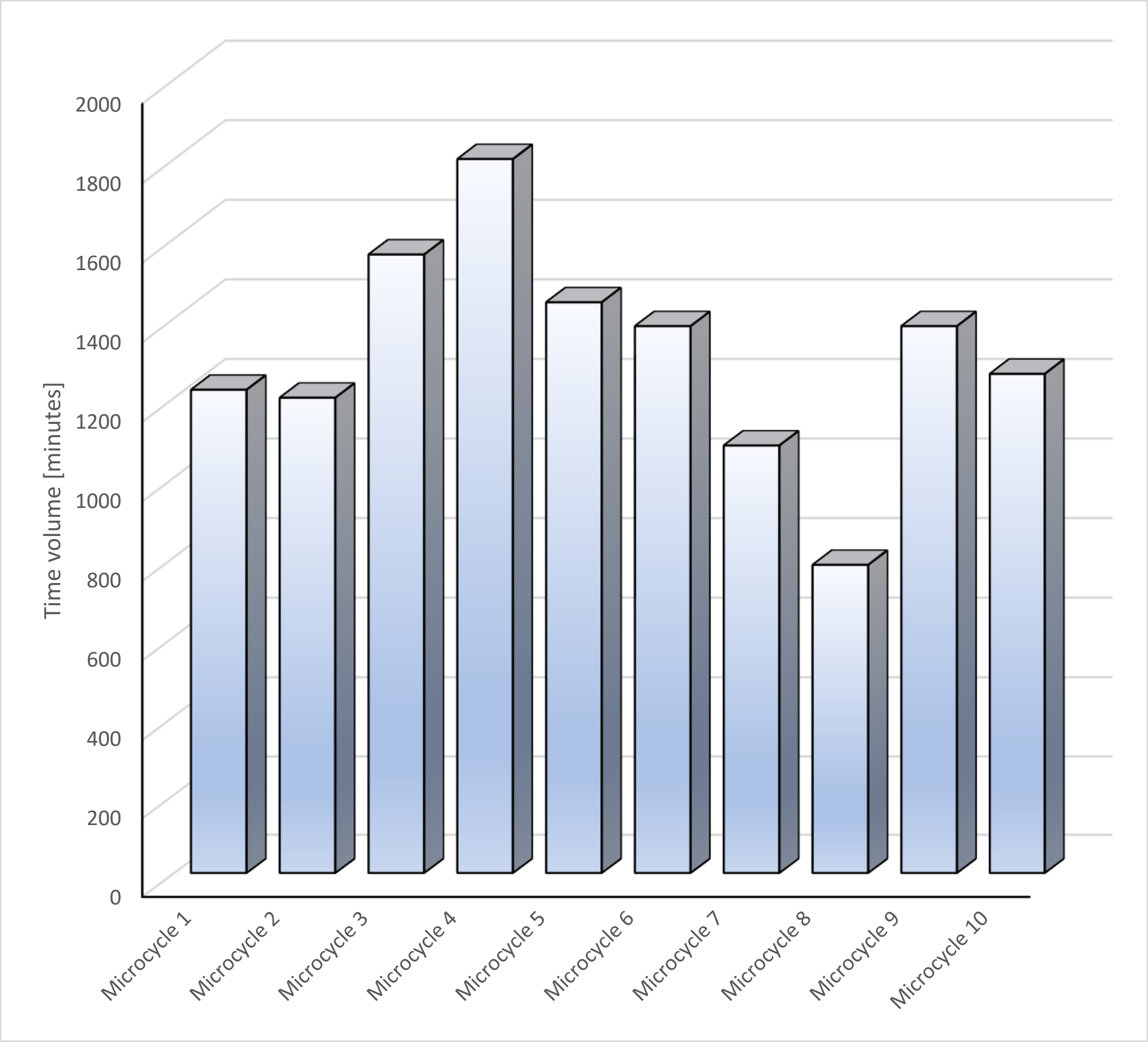

Figure S1. Time volume (minutes) in respective microcycles.

STRENGTH TRAINING

The amount of the loads applied by the team in strength training was totally 2903,869 tons (almost 3 million kilograms!). In each of the following microcycles, strength training in the players was included, while only its character changed (hypertrophy - endurance - dynamics - power).

On average, the players worked during the 10-week preparatory period with a load of 223 729 kg. The highest load was realized by the players in the 4th microcycle of the preparatory cycle, where the average load for the team was 380 081 kg, while the lowest was during the 8 microcycle- 177 878. The highest burden was recorded by an individual who performed work with a load of 252 344 kg, while the lowest was 210 814 kg of load.

As part of strength training, the most frequently used exercises were:

Multi-joint strength exercises

Deep squat

Squat 90

Squat on the crane

One-legged squat

Jefferson's squat

Deadlift with bent legs

Straight legs deadlift

A deadlift sumo with straight legs

Deadlift sumo with bent legs

Swallow with a griffin

Bending NN

Plea

Snatch

Extrusion on a flat

Squeezing on a slant

Dumbbell bench press on a flat

Dumbbell bench press on a slant

Flat heels

Leaflets on an oblique

Pulover

One-handed rowing

Rowing with both hands while lying forward

Front-lying dumbbell lift

Sideways lifting dumbbells while sitting

Sideways lifting dumbbells while standing

Squeezing dumbbells while sitting

Pulling up on the stick in the hold

Pulling up on a stick in the undercarriage

Extrusion on the NN machine

Extrusion on the N machine

Climbing on the toes of NN

Climbing on the toes of N

Climbing the landing

Preventive strength exercises

Butterfly

Pulling the bar down to the nape of the neck

Pulling the bar down to the chest

Pulling on the lower bar

Dumbbell bench press, sitting, "Arnold"

Pressing the bar while sitting on the crane

Bending N

Straightening LV

Straighten N

Olympic exercises

Plea of squeezing

Moving the bar overhead sideways

Sideways tearing

Table S2. The volume of training load (kilograms) for the team in respective microcycles.

Microcycle number	Kilograms
**Microcycle 1**	**358760**
**Microcycle 2**	**368418**
**Microcycle 3**	**302458**

**Microcycle 4**	**380081**
**Microcycle 5**	**332710**
**Microcycle 6**	**301518**
**Microcycle 7**	**246894**
**Microcycle 8**	**177878**
**Microcycle 9**	**223493**
**Microcycle 10**	**216268**
**Total**	**2903869**

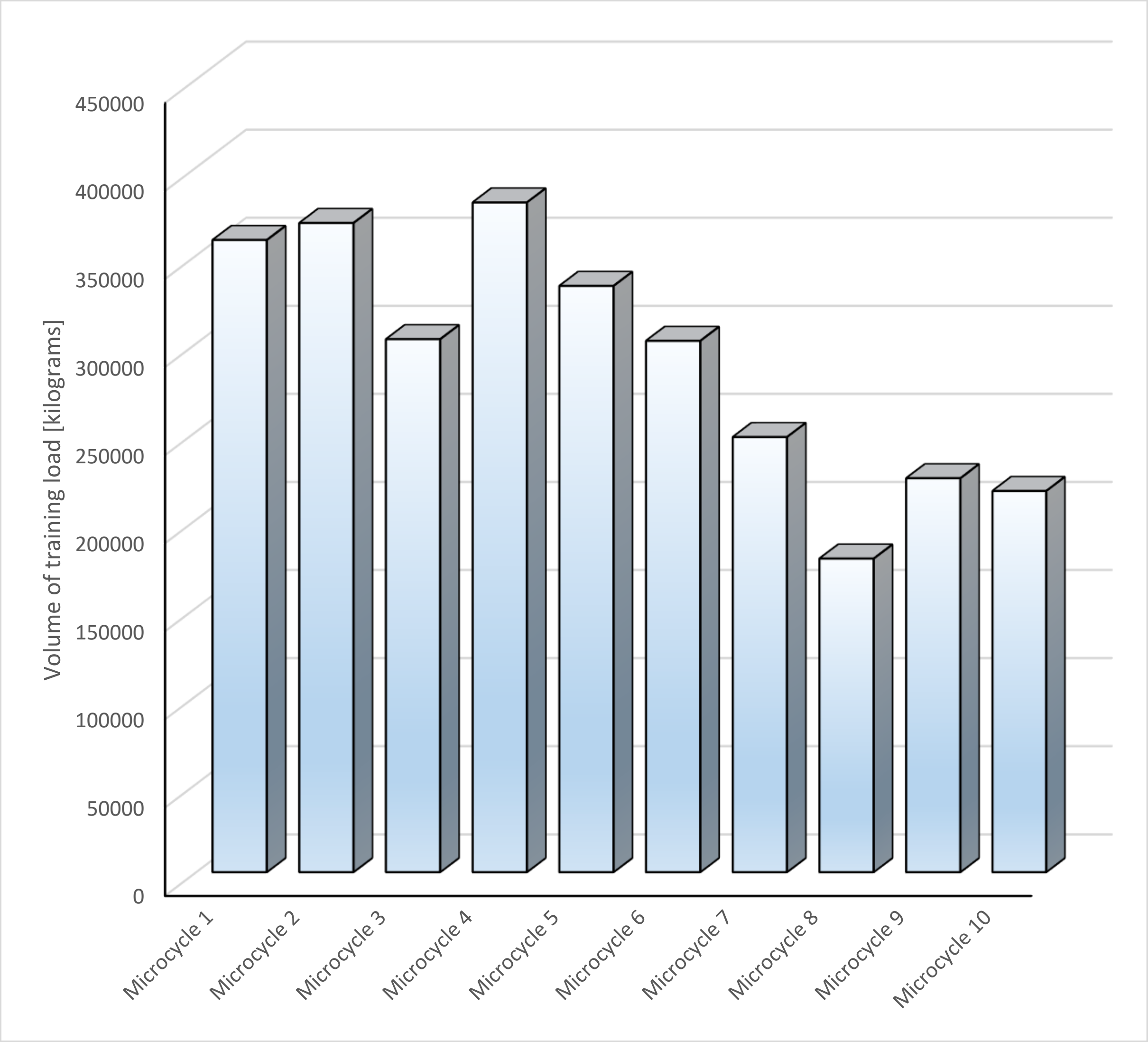

Figure. S2 The volume of training load (kilograms) for the team in respective microcycles

The distance covered by the players was determined on the basis of training plans and values measured with the Aktigraph WGT3X-BT accelerometer. In total, the team covered the distance of 362 745 km in preparation. As part of the cardiopulmonary adaptation, in the first 3 weeks, a trail running form was used - 1 week 5 km, 2 week 8, 3 week 8 km. The average distance covered in the following microcycles was 362.75 km. The lowest distance measured for single participant was 2285 meters, while the longest distance was 4273 meters. The average value of the distance covered by the competitor within one training unit was 3265 meters.

Table S3. Average distances covered by players during the preparation period (km)

Microcycle number	Kilometers
Microcycle 1	327,66
Microcycle 2	320,04
Microcycle 3	411,48
Microcycle 4	428,34
Microcycle 5	304,8
Microcycle 6	434,34
Microcycle 7	326,37
Microcycle 8	205,74
Microcycle 9	480,06
Microcycle 10	388,62
Total	3627,45

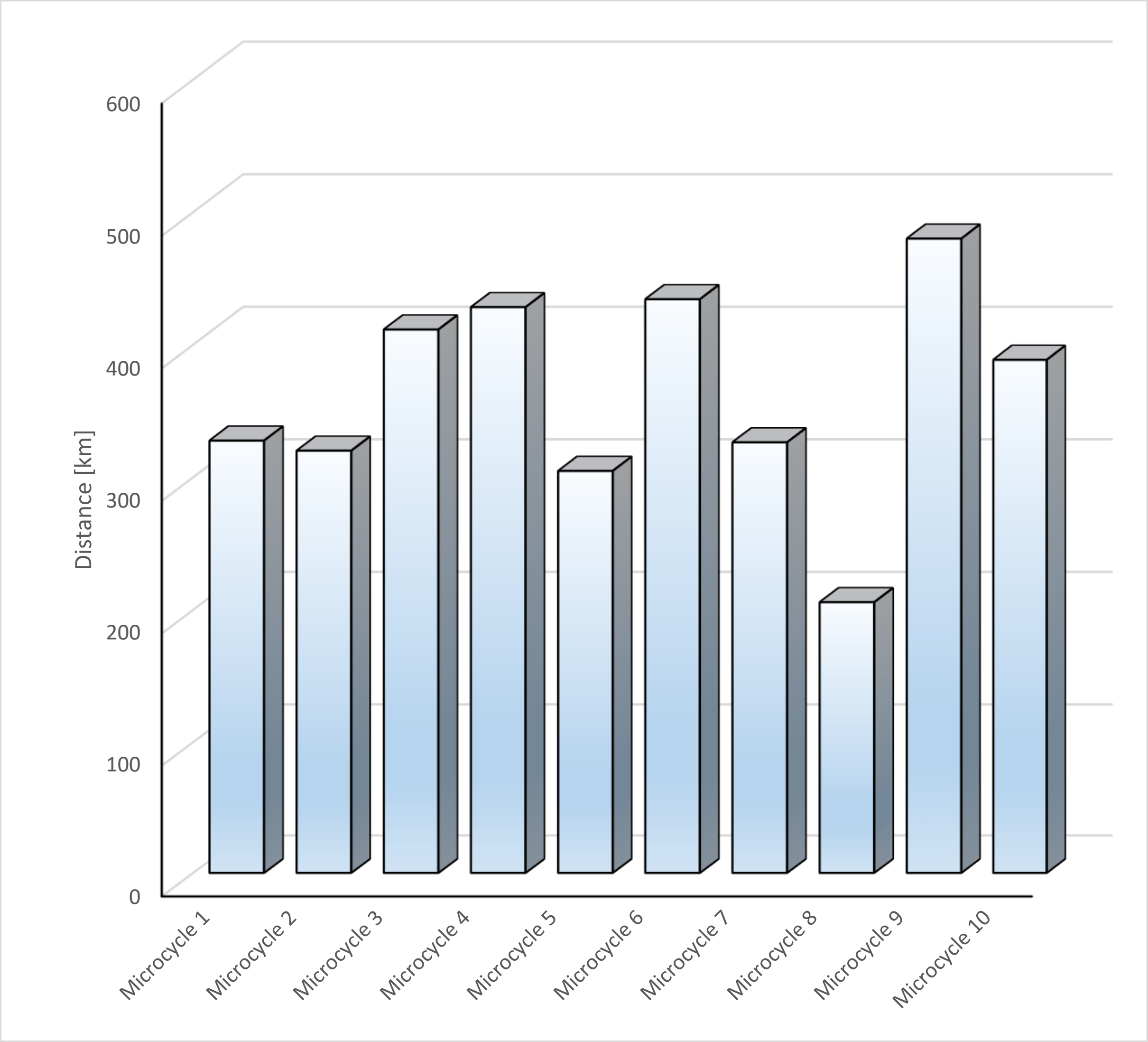

Figure. S3 Total distance (kilometers) covered by the team in respective microcycles

Detailed statistical description

Table S4. The Spearman's rank correlation between studied markers in the first training period (baseline).

	miR-223	miR-320a	miR-486	*IGF1R*	WEIGHT	BMI	BMR	FAT%	FAT MASS	FFM	TBW	CK	Cortisol
**miR-223**	1	**0.74** (p=0.006)	0.12	0.09	-0.39	-0.18	-0.26	-0.35	-0.52	-0.33	-0.33	-0.02	-0.2
**miR-320a**	**0.74** (p=0.006)	1	0.45	-0.1	-0.19	-0.39	-0.15	-0.08	-0.25	-0.15	-0.15	0.15	-0.28
**miR-486**	0.12	0.45	1	-0.48	0.3	-0.1	0.27	0.1	0.23	0.21	0.21	0.05	-0.35
IGF1R	0.09	-0.1	-0.48	1	-0.32	-0.31	-0.18	-0.31	-0.31	-0.23	-0.23	-0.34	-0.05

Statistically significant values of Spearman's rank correlation coefficients (rs) were bold and the p-values were provided. In cases without the p-values, the rs coefficient represents insignificant data.

Abbreviations: please refer to table 2.

Table S5. The Spearman's rank correlation between studied markers in the second training period.

	miR-223	miR-320a	miR-486	*IGF1R*	WEIGHT	BMI	BMR	FAT%	FAT MASS	FFM	TBW	CK	Cortisol
**miR-223**	1	-0.21	-0.03	-0.3	-0.5	-0.12	-0.48	-0.22	-0.31	-0.54	-0.54	0.21	-0.07
**miR-320a**	-0.21	1	**0.92** (p<0.001)	0.18	-0.13	-0.36	-0.1	-0.1	-0.2	-0.09	-0.09	-0.004	-0.5
**miR-486**	-0.03	**0.92** (p<0.001)	1	0.07	-0.16	-0.44	-0.17	-0.22	-0.26	-0.17	-0.17	-0.06	-0.57
IGF1R	-0.3	0.18	0.07	1	-0.21	-0.43	-0.21	-0.2	-0.16	-0.17	-0.17	-0.57	-0.07

Statistically significant values of Spearman's rank correlation coefficients (rs) were bold and the p-values were provided. In cases without the p-values, the rs coefficient represents insignificant data.

Abbreviations: please refer to table 2.

Table S6. The Spearman's rank correlation between studied markers in the third training period.

	**miR-223**	**miR-320a**	**miR-486**	***IGF1R***	**WEIGHT**	**BMI**	**BMR**	**FAT%**	**FAT MASS**	**FFM**	**TBW**	**CK**	**Cortisol**
**miR-223**	1	0.4	0	-0.3	-0.27	-0.13	-0.23	-0.22	-0.29	-0.23	-0.23	-0.19	0.33
**miR-320a**	0.4	1	**0.82** (p=0.001)	0.27	0.2	0.11	0.17	0.33	0.26	0.17	0.17	-0.15	-0.28
**miR-486**	0	**0.82** (p=0.001)	1	0.4	0.23	0.15	0.15	0.44	0.3	0.15	0.15	-0.01	-0.52
IGF1R	-0.3	0.27	0.4	1	-0.12	**-0.62** (p=0.032)	-0.06	-0.02	-0.03	-0.06	-0.06	-0.32	-0.44

Statistically significant values were bolded. Abbreviations: please refer to table 2.

Table S7. The Spearman's rank correlation between studied markers in the fourth training period.

	**miR-223**	**miR-320a**	**miR-486**	***IGF1R***	**WEIGHT**	**BMI**	**BMR**	**FAT%**	**FAT MASS**	**FFM**	**TBW**	**CK**	**Cortisol**
**miR-223**	1	0.34	-0.07	**-0.63** (p=0.028)	0.19	0.53	0.22	-0.04	-0.07	0.13	0.13	**0.59** (p=0.044)	-0.13
**miR-320a**	0.34	1	**0.8** (p=0.002)	0.15	0.24	0.007	0.24	0.19	0.19	0.26	0.18	0.38	-0.15
**miR-486**	-0.07	**0.8** (p=0.002)	1	0.29	0.12	-0.18	0.1	0.15	0.22	0.13	0.06	0	0.05
IGF1R	**-0.63** (p=0.028)	0.15	0.29	1	0.13	-0.28	0.18	0.29	0.34	0.17	0.16	-0.29	-0.06

Statistically significant values were bolded. Abbreviations: please refer to table 2.

Table S8. The multiple linear regression analysis using insulin-like growth factor 1 receptor gene expression levels as outcome .

The expression level of *IGF1R* was used as dependent variable. $R = 0.51$; $R^2 = 0.26$ and R^2 (adjusted) = 0.17						
**Variable name**	b*	Standard error from b*	b	Standard error from b	T	p-value
Intercept			**3.42298**	**1.541206**	**2.22097**	**0.031936**
**miR-320a**	**0.608751**	**0.260381**	**2.89478**	**1.238186**	**2.33792**	**0.024351**
**miR-223**	-0.394823	0.203758	-1.62701	0.839659	-1.93771	0.059567
**miR-486**	-0.088589	0.210341	-0.32774	0.778165	-0.42117	0.675833
**Cortisol**	-0.089181	0.154977	-0.02834	0.049242	-0.57544	0.568136
**CK**	**-0.317921**	**0.139299**	**-0.00734**	**0.003217**	**-2.28229**	**0.027731**

Abbreviations: please refer to table 2. Statistically significant values were bolded.

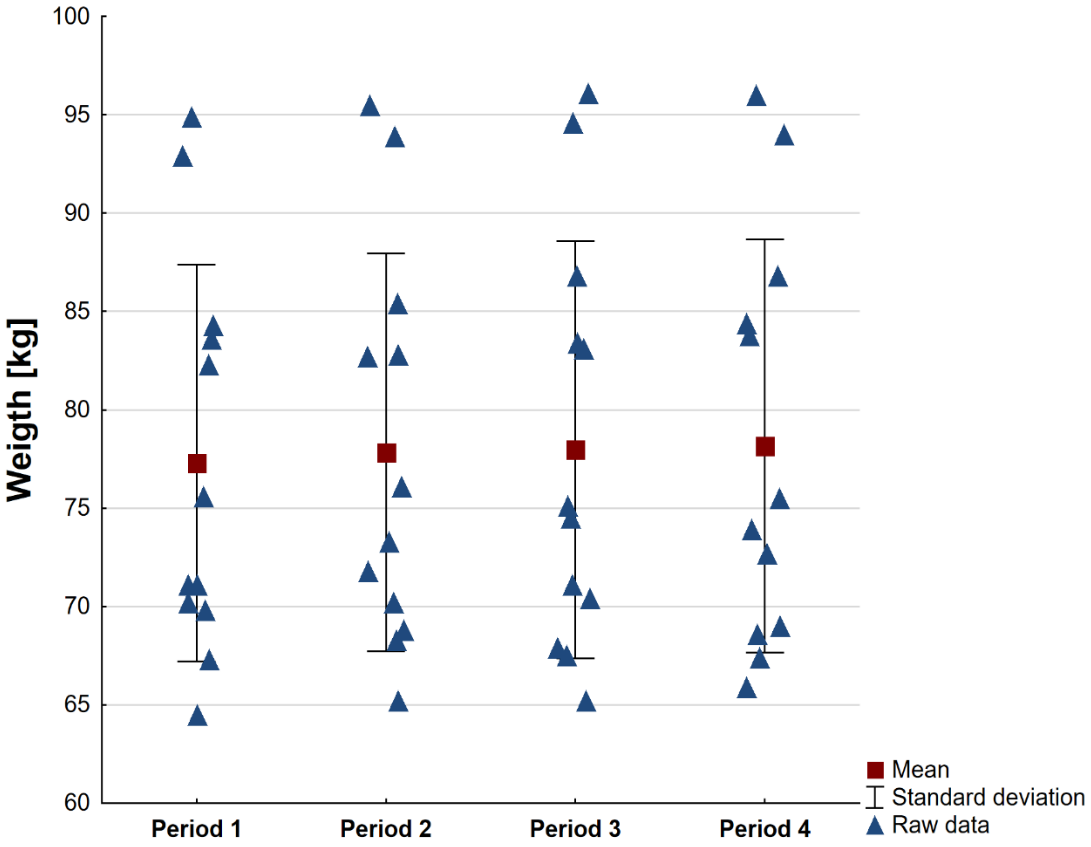

Figure S4. The changes in weight between training periods.

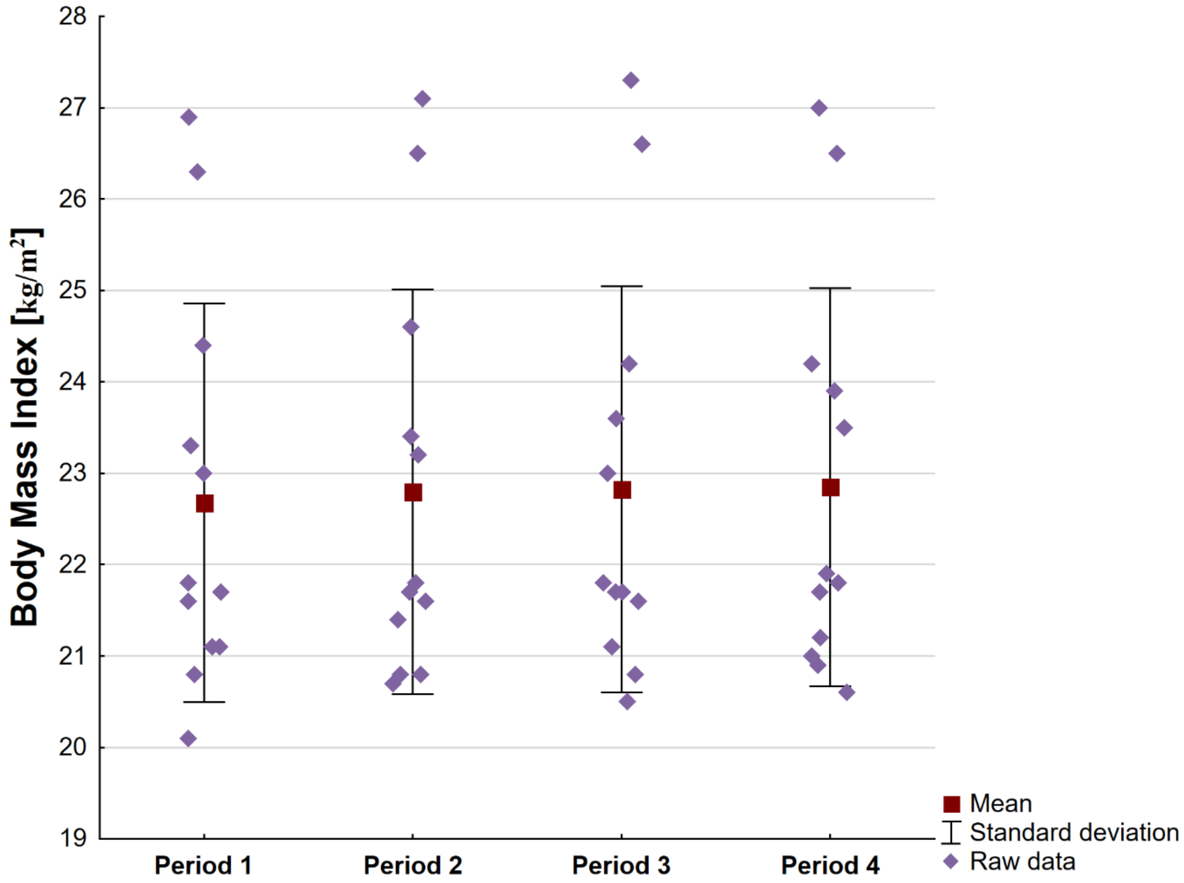

Figure S5. The changes in body mass index between training periods.

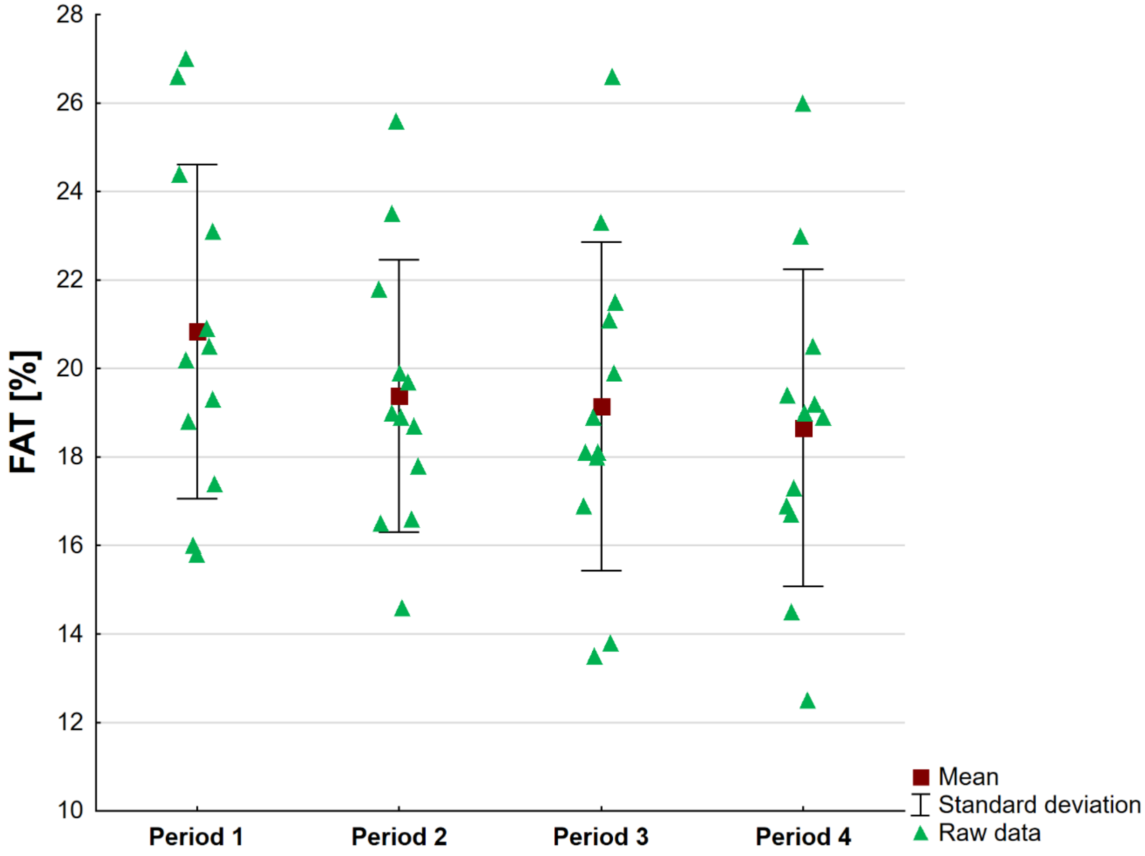

Figure S6. The changes in percentage of fat between training periods.

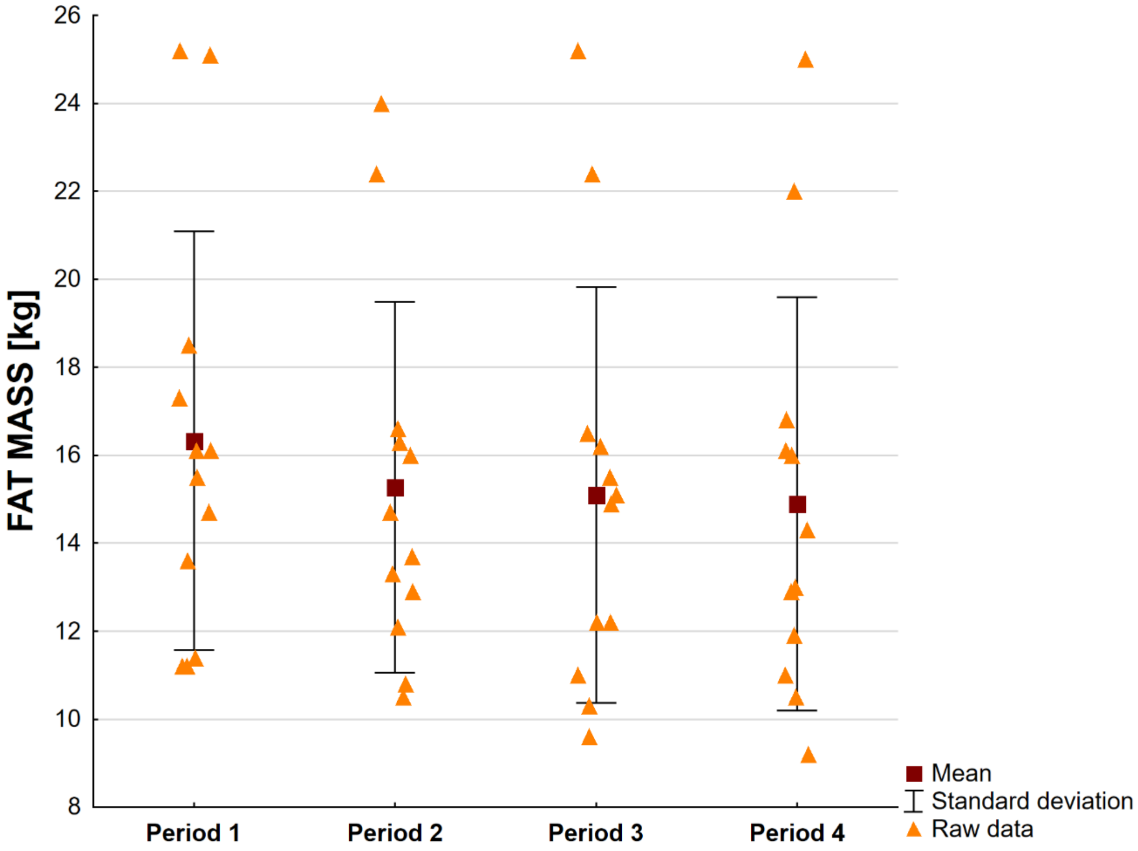

Figure S7. The changes in fat mass between training periods.

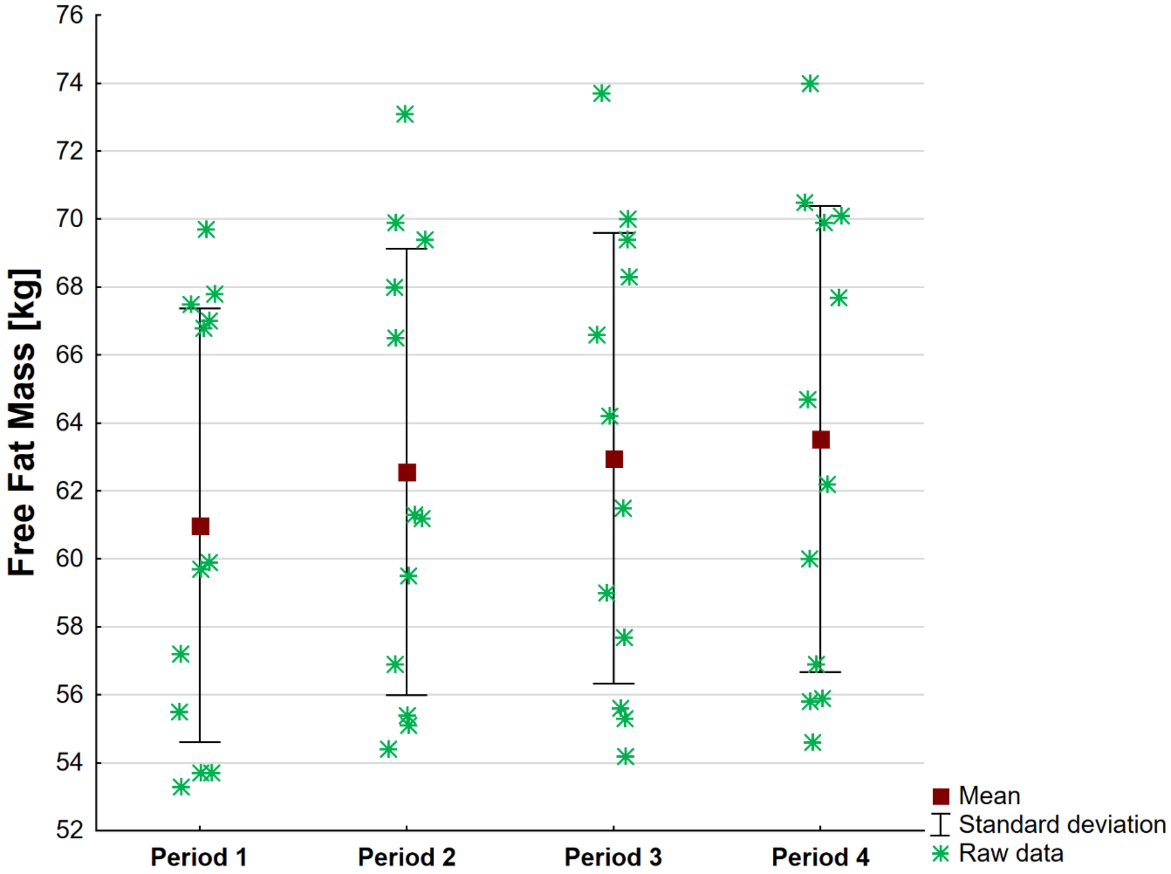

Figure S8. The changes in fat-free mass between training periods.

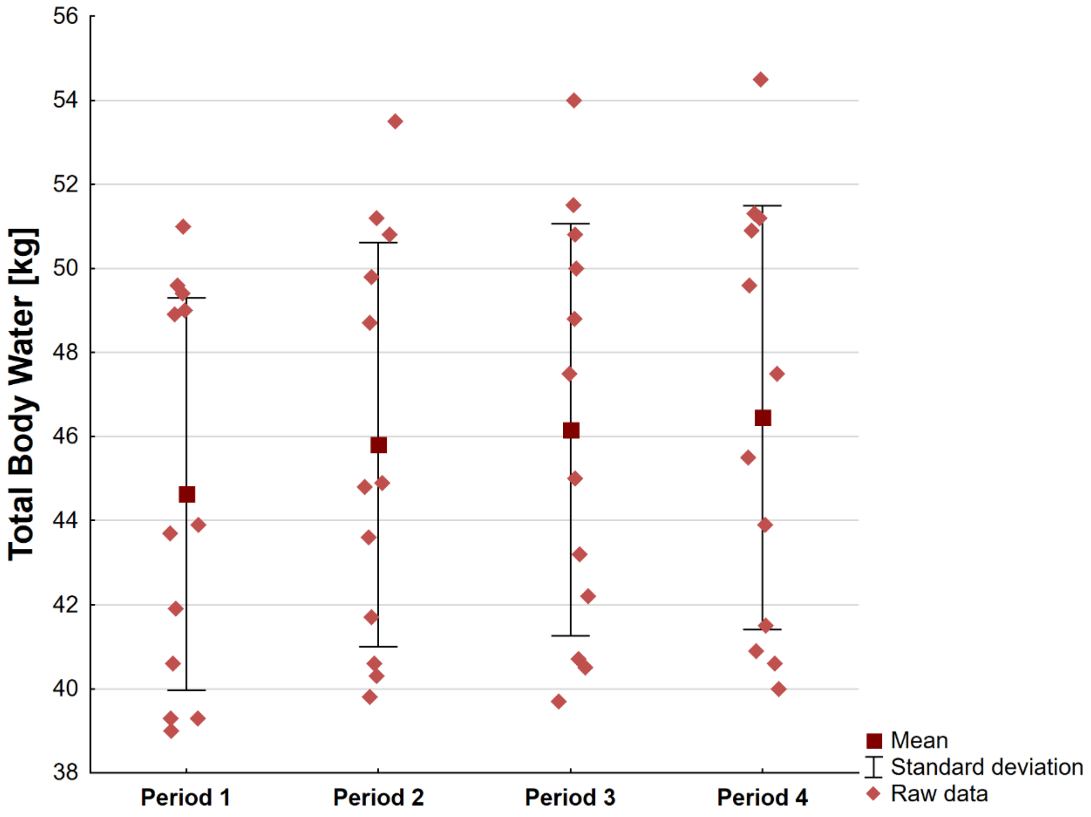

Figure S9. The changes in total body water between training periods.

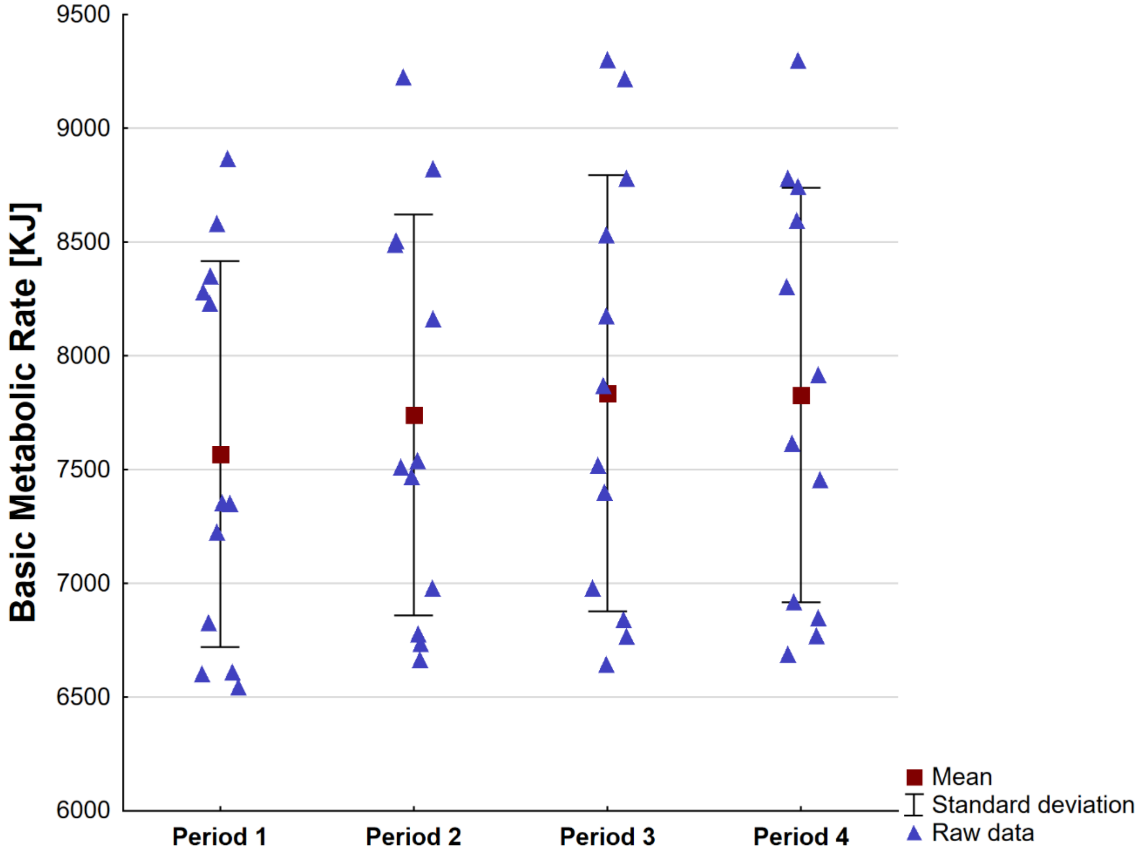

Figure S10. The changes in basic metabolic rate between training periods.

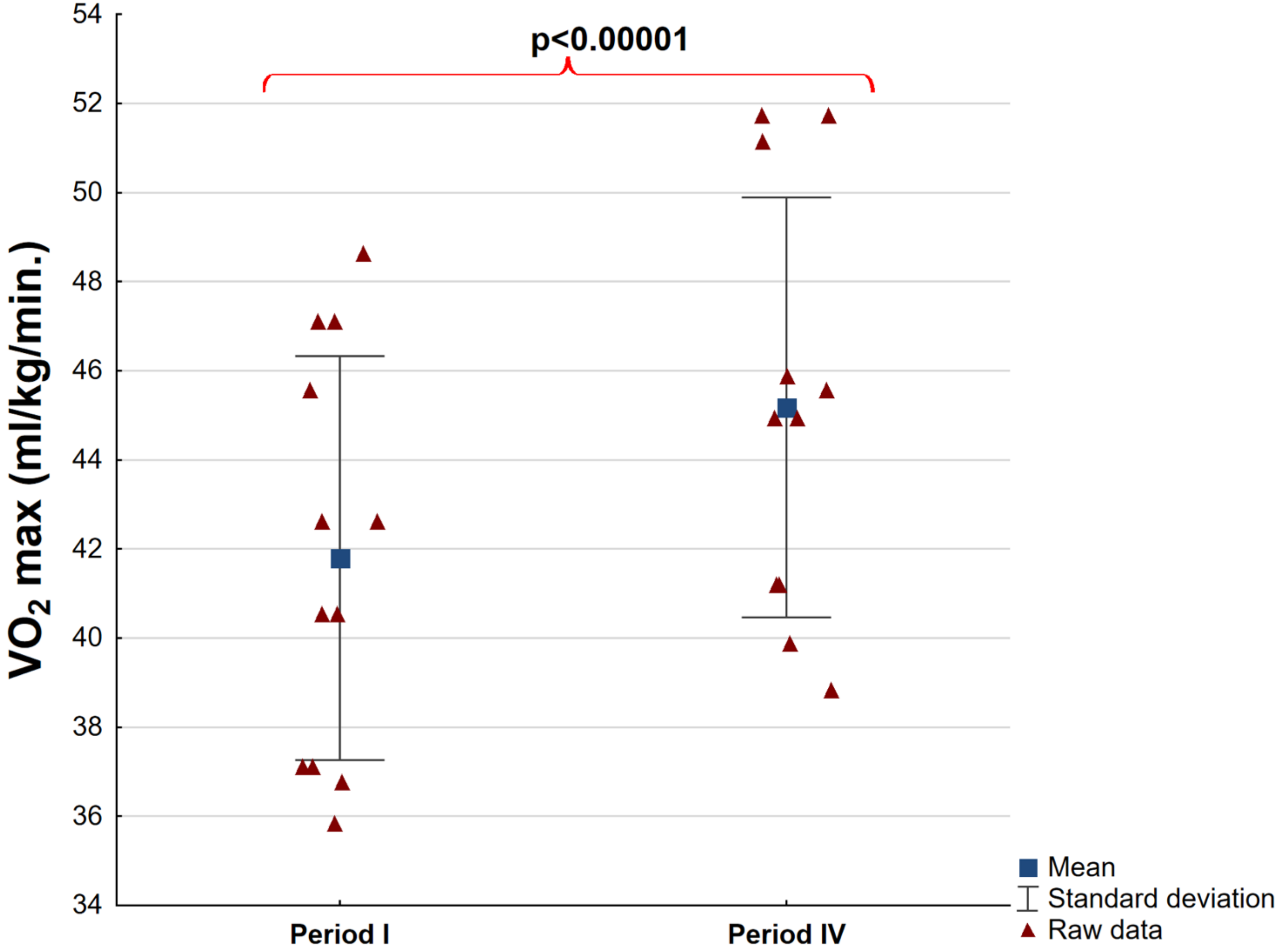

Figure S11. The changes in VO_2 max rate between first and last training period.

(Statistical differences were calculated by the t-test for dependent samples)
